# *Glutathione S-transferase theta 1* (*GSTT1*) deletion polymorphism and susceptibility to head and neck carcinoma: a systematic review with five analyses

**DOI:** 10.1186/s12885-024-12618-7

**Published:** 2024-07-22

**Authors:** Sepehr Sadafi, Parsia Choubsaz, Seyed Mohammad Mohyeddin Kazemeini, Mohammad Moslem Imani, Masoud Sadeghi

**Affiliations:** 1 Molecular Pathology Research Center, Imam Reza Hospital, Kermanshah University of Medical Sciences, Kermanshah, Iran; 2 Clinical Research Development Center, Imam Reza Hospital, Kermanshah University of Medical Sciences, Kermanshah, Iran; 3 Department of Orthodontics, School of Dentistry, Shahid Beheshti University of Medical Sciences, Tehran, 1983963113 Iran; 4 Department of Biology, Science and Research Branch, Islamic Azad University, Tehran, 1477893855 Iran; 5 Department of Orthodontics, Kermanshah University of Medical Sciences, Kermanshah, Iran; 6 Medical Biology Research Center, Health Technology Institute, Kermanshah University of Medical Sciences, Kermanshah, Iran

**Keywords:** Head and neck carcinoma, Genotype, Polymorphism, GSTT1, Meta-analysis

## Abstract

Glutathione S-transferase theta 1 (GSTT1) enzyme plays a key role in the neutralization of electrophilic compounds such as carcinogens. Herein, we aimed to evaluate *GSTT1* deletion polymorphism and susceptibility to head and neck carcinoma (HNC) according to 107 articles in a systematic review with five analyses. The databases of PubMed/Medline, Web of Science, Scopus, and Cochrane Library from the beginning of each database until June 21, 2023, with no restrictions to identify pertinent articles. The RevMan 5.3 software was used to calculate the effect sizes, which were displayed as the odds ratio (OR) along with a 95% confidence interval (CI). Both the publication bias and sensitivity analyses were performed using the CMA 3.0 software. A trial sequential analysis (TSA) was conducted. Of the 1966 records retrieved from four databases, 107 articles were included in the analysis. The combined analysis revealed that the pooled OR was 1.28 (95% CI: 1.14 to 1.44; *p*-value < 0.0001). The pooled OR was highest in mixed ethnicity. Nasopharyngeal cancer had the highest OR (1.84), followed by oral cancer (OR = 1.20), and laryngeal cancer (OR = 1.17). Studies with less than 200 samples had a higher OR compared to those with 200 or more samples. The studies with a quality score of 7 or more had a higher OR compared to those with a score of less than 7. When both age and sex are considered, while the OR of 1.42 is significant, the high heterogeneity suggests caution in interpreting these results. There is no evidence of publication bias. TSA reported that the study does not have sufficient statistical power. This comprehensive meta-analysis revealed a significant association between the *GSTT1* null genotype and an increased risk of HNC, with variations based on factors such as ethnicity, cancer type, sample size, control source, and quality score.

**Supplementary Information:**

The online version contains supplementary material available at 10.1186/s12885-024-12618-7.

## Introduction

Head and neck cancer (HNC) is a group of cancers that starts within the nose, mouth, throat, sinuses, larynx, or salivary glands (1, 2). According to the most recent GLOBOCAN estimates from 2020, HNC ranks as the seventh most prevalent cancer worldwide. It is responsible for approximately 890,000 new cases annually, representing roughly 4.5% of all cancer diagnoses globally (3). Additionally, HNC leads to approximately 450,000 deaths each year, accounting for approximately 4.6% of total cancer-related deaths worldwide (3). Men are more prone to HNC than women, irrespective of their alcohol consumption or tobacco use habits. This gender disparity in the incidence of HNC becomes particularly noticeable in individuals in their 60s. The lower regions of the upper aerodigestive tract, such as the larynx and hypopharynx, are the most commonly affected areas (4). Although tobacco and alcohol are the main risk factors for the development of HNC, a significant correlation has been observed between a subset of HNC and the human papillomavirus in epidemiological studies (5). A number of single nucleotide polymorphisms can associate with the risk of HNC reported in recent meta-analyses (6–10).

*Glutathione S-transferase theta 1 (GSTT1)* gene produces an enzyme that plays a key role in the neutralization of electrophilic compounds. These compounds include carcinogens, therapeutic drugs, environmental toxins, and by-products of oxidative stress (11). *GSTT1* is located at 22q11.23 with a 480 bp fragment using specific primers (12, 13). and the *GSTT1* null genotype, which results from a homozygous deletion of the *GSTT1* gene, leads to a lack of enzyme activity (14).

However, the association between *GSTT1* deletion polymorphism and HNC remains unclear due to inconsistent findings among studies. Some studies have reported a significant association (15–17), while others have found no such link (18–21). These discrepancies could be due to differences in study design, sample size, population characteristics, or methods of genotyping.

Several meta-analyses (22–36) reported the association of *GSTT1* deletion polymorphism and HNC susceptibility. Three meta-analyses (24, 28, 32) were published after 2015 and all three meta-analyses just reported *GSTT1* deletion polymorphism in oral cancer. The last meta-analysis (32) included 36 studies.

This systematic review aimed to provide a more definitive answer to this question by combining the results of 107 articles (50 studies reported oral cancer).

## Materials and methods

### Study design and registration

The meta-analysis was conducted by the protocols of the Preferred Reporting Items for Systematic Reviews and Meta-Analyses (PRISMA) (37). The question posed in terms of PICO (population, intervention, comparison, and outcome) was (38): Does the deletion polymorphism of *GSTT1* associate with the susceptibility to HNC in case-control studies? (Population (P): Patients with HNC. Intervention (I): *GSTT1* deletion polymorphism. Comparison (C): Control subjects (non-HNC individuals). Outcome (O): Correlation with susceptibility to HNC in case-control studies). The study has not registered in any database.

### Identification of articles

An exhaustive search was carried out by one author (M.S.) in the databases of PubMed/Medline, Web of Science, Scopus, and Cochrane Library from the beginning of each database until June 21, 2023, with no restrictions to identify pertinent articles. The titles/abstracts of the articles were evaluated by the same author (M.S), who also downloaded the full texts of the articles that satisfied the eligibility criteria. The search strategy encompassed: (“Glutathione S-transferase” OR “GSTT1” OR “GST”) AND (“head and neck” OR “oral” OR “OSCC” OR “tongue” OR “mouth” OR “HNSCC“ OR “nasopharyngeal” OR " oropharyngeal " OR “nasopharynx” OR “salivary gland” OR “hypopharyngeal” OR “pharyngeal” OR “pharynx” OR “oral cavity” OR “hypopharynx” OR “laryngeal” OR “larynx”) AND (“carcinoma*” OR “tumor*” OR “cancer*” OR “neoplasm*”) AND (“allele*” OR “variant*” OR “genotype*” OR “gene*” OR “polymorphism*”). To ensure no relevant study was overlooked, the reference lists of the articles were also scrutinized. The search and selection process was re-verified by another author (M.M.I.). In case of any disagreement between the two authors, a third author (M.S.) resolved it.

### Inclusion and exclusion criteria

The inclusion criteria were as follows: (1) Studies of the case-control type that examined *GSTT1* deletion polymorphism in patients with HNC and control subjects. (2) HNC was diagnosed clinically and pathologically. (3) Patients with HNC did not have any other systemic diseases and controls were either healthy or free from cancer. Conversely, the exclusion criteria included: review articles, meta-analyses, systematic reviews, articles that had incomplete data or lacked a control group, studies conducted on animals, conference papers, comment papers, duplicate studies, book chapters, studies that included controls with the disease, and studies that included cases under treatment.

### Data summary

The information from the studies incorporated into the meta-analysis was independently gathered by two authors (M.S. and S.S.). Any disagreements were settled through discussion.

### Quality evaluation

One author (M.S) performed the quality scoring using the Newcastle-Ottawa Scale (NOS) tool (39). This tool evaluates a study based on three broad perspectives: the selection of the study groups (4 scores), the comparability of the groups (2 scores), and the ascertainment of either the exposure or outcome of interest (3 scores) for case-control studies. The maximum possible score is nine, and a score of ≥ 7 is considered to be of high quality. Another author (N.K.) re-checked the scores. Disagreement between the authors was resolved by a short discussion.

#### Statistical analyses

The Review Manager 5.3 (RevMan 5.3) software was used to calculate the effect sizes, which were displayed as the odds ratio (OR) along with a 95% confidence interval (CI) for the prevalence of the null genotype of *GSTT1* polymorphism in HNC patients and controls. The significance of the pooled OR was determined using the Z-test, with a two-sided *p*-value less than 0.05 deemed significant. A random-effects model (40) was employed if P_heterogeneity_ was < 0.10 (I^2^ > 50%), indicating significant heterogeneity. If the heterogeneity was not significant, a fixed-effect model (41) was applied.

A subgroup analysis was conducted to ascertain whether the combined effect sizes in these subgroups differed significantly from one another. Furthermore, a meta-regression analysis using a random-effects model was carried out to illustrate a linear correlation between auxiliary variables in the study and the effect size.

The extent of publication bias was assessed using the funnel plot and Egger’s regression test. The possibility of publication bias was evaluated using Begg’s funnel plot and Begg’s test, and the level of asymmetry was tested with Egger’s test. The p-values from both Egger’s and Begg’s tests were obtained, and a 2-sided p-value less than 0.10 indicated the existence of publication bias. In terms of sensitivity analysis, both “one-study-removed” (This is done to determine if any single study has a disproportionate impact on the overall estimate.) and “cumulative” (This is done to assess the impact of each additional study on the overall estimate.) analyses were employed to assess the stability and consistency of the pooled SMDs. Both the publication bias and sensitivity analyses were performed using the Comprehensive Meta-Analysis version 3.0 (CMA 3.0) software.

The Radial plot, also known as the Galbraith plot, was designed using the NCSS 2021 version 21.0.2 software. This plot displays the z-statistic (obtained by dividing by the standard error) on the vertical axis and the weight measurement on the horizontal axis (42). A *p*-value less than 0.05, indicates statistically significant heterogeneity.

To mitigate the risk of false-positive or negative conclusions from meta-analyses (43), a TSA was conducted using TSA software (version 0.9.5.10 beta) from the Copenhagen Trial Unit, Centre for Clinical Intervention Research, Rigshospitalet, Copenhagen, Denmark (44). TSA allows for the testing of a futility threshold to establish a result of no effect before reaching the necessary information size. The required information size (RIS) was computed with an alpha risk of 5%, a beta risk of 20%, and a two-sided boundary type. Heterogeneity (D2) was evaluated for the prevalence of the null genotype of *GSTT1* polymorphism in HNC patients and controls. If the Z-curve reached the RIS line or traced the boundary line or futility area, it suggested that the studies included a sufficient number of cases and that the conclusions were reliable. If not, it indicated that the information available was insufficient and additional data was required.

## Results

### Study selection

**Fig. 1:**
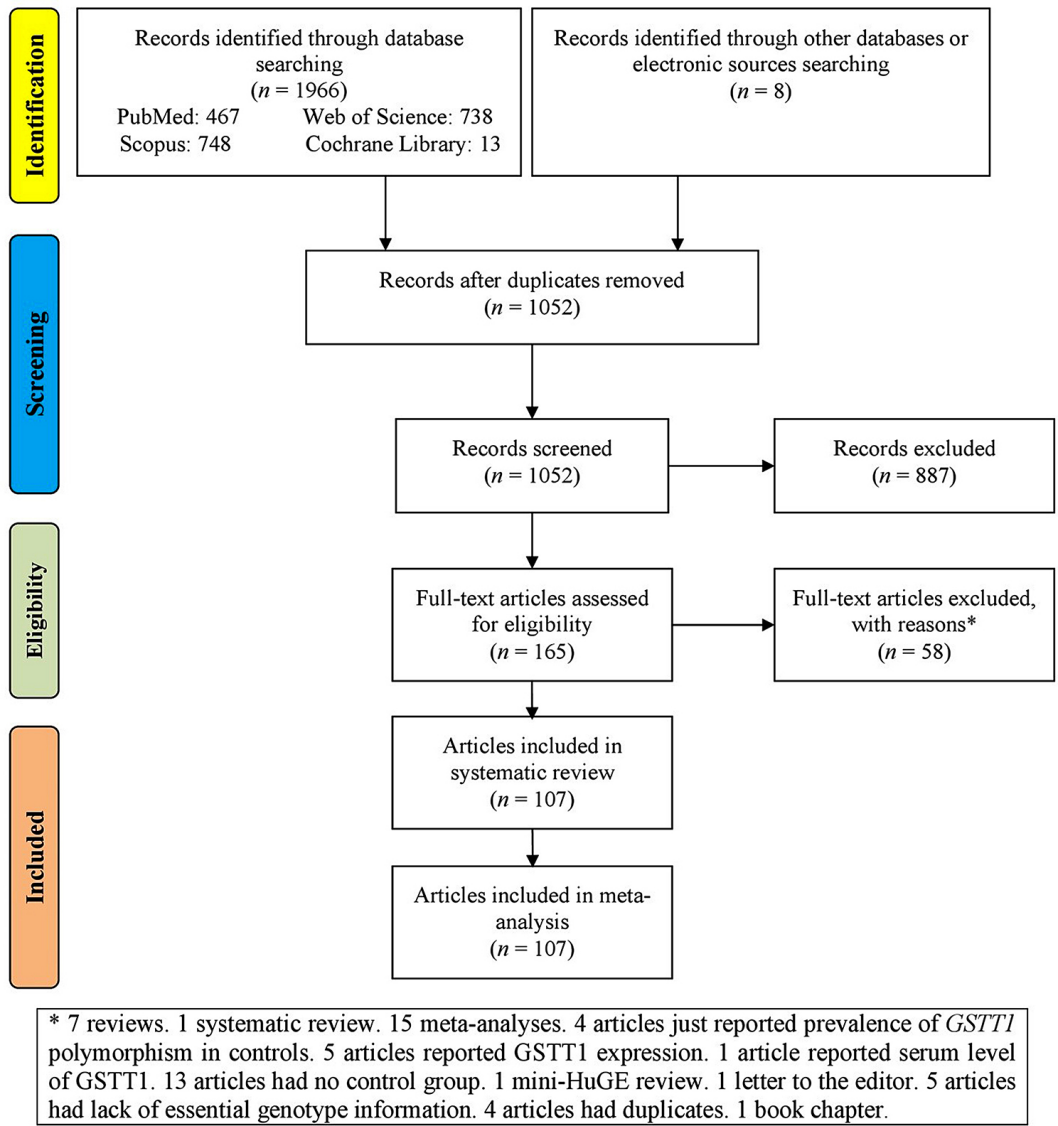
Flowchart of study selection for systematic review and meta-analysis

A total of 1966 records were initially retrieved from four databases, along with 8 records from other electronic sources (Fig. 1). After the removal of duplicates, 1052 records remained and were screened. Of these, 887 records were deemed irrelevant and subsequently removed. This left 165 full-text articles that met the eligibility criteria. However, 58 of these were excluded for various reasons. Ultimately, 107 full-text articles were included in the analysis.

### Study’s characteristics

Table 1 presents a comprehensive list of 107 articles (15–21, 45–142) including 109 studies conducted on the null genotype of *GSTT1* polymorphism in HNC patients and controls. The studies span multiple countries and ethnicities, with a variety of cancer types and control sources. Each study includes the number of cases and controls, with some studies matching controls based on age and sex. The quality score of each study is also provided, offering insight into the reliability of the data.

**Table 1:** Characteristics of the studies

First author, publication year	Country	Ethnicity	Cancer type	Control source	Genotyping method	Number of cases/controls	Control matching (age and sex)	Case		Control			Quality score
								Present	Null	Present	Null		
Acar, 2006 (45)	Turkey	Caucasian	LC	PB	PCR	110/197	None	77	33	166	31		7
Alamgir, 2022 (15)	Pakistan	Asian	OC	HB	PCR-RFLP	123/62	None	105	18	60	2		6
Amtha, 2009 (46)	Indonesia	Asian	OC	HB	PCR multiplex	81/162	Both	44	37	95	67		8
Anantharaman, 2007 (47)	India	Asian	OC	PB	PCR	451/727	Age	411	45	612	114		9
Anantharaman, 2011 (48)	India	Asian	OC	PB	PCR	592/788	Both	547	45	674	114		7
Bathi, 2009 (49)	India	Asian	OC	HB	PCR	30/60	Both	10	20	15	45		9
Ben Chaaben, 2015 (50)	Tunisia	Mixed	NPC	PB	PCR-RFLP	245/309	None	123	122	222	87		6
Bendjemana, 2006 (51)	Algeria and Tunisia	Mixed	NPC	PB	PCR multiplex	45/100	None	36	9	84	16		7
Bendjemana, 2014 (52)	Tunisia	Mixed	NPC	HB	PCR multiplex	132/200	Both	100	32	160	40		8
Biselli, 2006 (53)	Brazil	Mixed	HNC	HB	PCR multiplex	60/60	Both	40	20	46	14		8
Boccia, 2008 (54)	Italy	Caucasian	HNC	HB	PCR-RFLP	210/245	Both	162	48	187	58		8
Buch, 2002 (55)	India	Asian	OC	PB	PCR	297/450	None	243	54	395	55		7
Buch, 2008 (56)	USA	Mixed	OC	HB	PCR	195/414	Both	128	67	292	122		7
Cabelguenne, 2001 (57)	France	Caucasian	HNC	HB	PCR	162/264	Both	73	89	213	51		8
Capoluongo, 2007 (58)	Italy	Caucasian	HNC	HB	PCR	100/100	Both	65	35	69	31		8
Cha, 2007 (47)	Korea	Asian	OC	PB	PCR-RFLP	72/209	None	35	37	86	123		6
Chatterjee, 2010 (59)	India	Asian	OC	PB	PCR	102/100	Both	98	4	94	6		8
Chatzimichalis, 2010 (60)	Greece	Caucasian	LC	PB	PCR	88/102	None	67	21	71	31		7
Chen, 2010 (61)	Taiwan	Asian	OC	PB	PCR-RFLP	164/274	Both	77	87	122	152		8
Cheng, 1999 (62)	USA	Mixed	HNC	PB	PCR	162/315	Both	109	53	260	55		9
Cheng, 2003 (18)	Taiwan	Asian	NPC	PB	PCR-RFLP	316/336	Both	156	160	162	174		8
Choudhury, 2015 (63)	India	Asian	HNC	PB	PCR-RFLP	180/240	Both	115	65	162	78		9
Cornean, 2022 (64)	Romania	Caucasian	LC	HB	PCR-RFLP	92/101	Sex	61	31	81	20		8
D’ Mello, 2016 (16)	India	Asian	OC	HB	PCR	30/25	None	8	22	2	23		6
Deakin, 1996 (65)	England	Caucasians	OC	HB	PCR	34/509	None	30	4	415	94		6
Deng, 2004 (66)	China	Asian	NPC	HB	PCR	91/135	None	37	54	80	55		6
Deng, 2005 (67)	China	Asian	NPC	PB	PCR	127/207	Both	48	79	122	85		9
Dong, 2016 (68)	China	Asian	OC	PB	PCR	750/750	Both	395	355	598	152		9
Drummond, 2005 (17)	Brazil	Mixed	OC	PB	PCR	87/81	Both	14	73	47	34		9
Evans, 2004 (69)	USA	Caucasian	HNC	PB	PCR	283/208	None	229	54	150	58		6
Firigato, 2019 (70)	Brazil	Mixed	HNC	HB	PCR	617/447	Both	489	128	349	98		7
Firigato, 2022 (71)	Brazil	Mixed	OC	HB	PCR	234/421	Both	196	38	326	95		7
Gajecka, 2005 (72)	Poland	Caucasian	LC	HB	PCR-RFLP	290/316	Sex	236	54	255	61		6
Gattás, 2006 (73)	Brazil	Mixed	HNC	HB	PCR multiplex	103/102	Both	78	25	84	18		8
Gaudet, 2004 (74)	USA	Mixed	HNC	HB	PCR	149/180	Both	122	27	158	22		8
Goloni-Bertollo, 2006 (75)	Brazil	Mixed	HNC	HB	PCR multiplex	45/55	None	34	11	47	8		6
Gronau, 2003 (76)	Germany	Caucasian	HNC	PB	PCR	187/139	Both	157	30	118		21	9
Gronau, 2003b (77)	Germany	Caucasian	OC	HB	PCR	73/136	None	62	11	117	19		6
Guo, 2008 (78)	China	Asian	NPC	PB	PCR multiplex	338/585	None	174	164	316	269		6
Hanna, 2001 (79)	USA	Mixed	LC	HB	PCR	20/20	Both	17	3	16	4		8
Harth, 2008 (80)	Germany	Caucasian	HNC	HB	PCR-RFLP	312/300	None	248	64	239	61		6
Hatagima, 2008 (81)	Brazil	Mixed	OC	HB	PCR-RFLP	231/212	Both	182	49	164	48		8
Hong, 2000 (82)	Korea	Asian	LC	PB	PCR	82/63	Sex	35	47	40	23		8
Huang, 2006 (83)	China	Asian	OC	HB	PCR	87/87	None	40	47	45	42		6
Hung, 1997 (84)	Taiwan	Asian	OC	PB	PCR	41/123	Age	17	24	58	65		8
Jahnke, 1995 (85)	Germany	Caucasian	LC	HB	PCR	169/145	None	133	36	127	18		5
Jahnke, 1996 (86)	Germany	Caucasian	LC	HB	PCR	269/216	None	213	56	188	28		6
Jaskula-Sztul, 1998 (87)	Poland	Caucasian	LC	PB	PCR	171/180	None	141	30	141	39		7
Jiang, 2011 (88)	China	Asian	NPC	HB	PCR-CTPP	182/366	Both	62	120	186	180		8
Jourenkova, 1998 (89)	France	Caucasian	LC	HB	PCR multiplex	129/172	Both	104	25	145	27		8
Jourenkova-Mironova, 1999 (90)	France	Caucasian	HNC	HB	PCR-RFLP	121/172	Both	95	25	145	27		8
Karen-Ng, 2011 (91)	Malaysia	Asian	OC	HB	PCR multiplex and PCR-RFLP	115/116	None	69	46	68	48		6
Katiyar, 2020 (92)	India	Asian	HNC	PB	PCR	1250/1250	Sex	933	317	1005	245		8
Katoh, 1999 (93)	Japan	Asian	OC	PB	PCR	92/147	None	48	44	72	75		7
Kietthubthew, 2001 (94)	Thailand	Asian	OC	PB	PCR	53/53	Both	35	18	28	25		9
Ko, 2001 (19)	Germany	Caucasian	HNC	PB	PCR	312/300	None	238	62	239	61		7
Kondo, 2009 (95)	USA	Mixed	OC	HB	PCR multiplex	166/511	Sex	120	46	406	105		8
Krüger, 2015 (96)	Germany	Caucasian	OC	PB	Real-time PCR	100/93	None	78	22	76	17		7
Leme, 2010 (97)	Brazil	Mixed	HNC	HB	PCR	100/100	Sex	48	52	41	59		7
Li, 2009 (98)	China	Asian	LHC	PB	PCR	76/76	None	32	44	37	39		7
Liao, 2005 (99)	China	Asian	NPC	HB	PCR	80/72	None	30	50	40	32		6
Liu, 2005 (100)	Taiwan	Asian	OC	HB	PCR	114/100	Both	63	51	63	37		8
Losi-Guembarovski, 2008 (101)	Brazil	Mixed	OC	PB	PCR-RFLP	91/81	Age	61	30	58	23		8
Lourenço, 2011 (102)	Brazil	Mixed	HNC	HB	PCR-RFLP	142/142	Sex	106	36	115	27		7
Madhulatha, 2018 (103)	India	Asian	OC	PB	PCR	25/25	None	16	9	20	5		7
Maniglia, 2020(104)	Brazil	Mixed	HNC	HB	PCR-RFLP	197/214	None	140	57	102	112		6
Marchioni, 2011 (105)	Brazil	Mixed	HNC	HB	PCR multiplex	103/101	Both	78	25	83	18		8
Masood, 2011 (106)	Pakistan	Asian	OC	HB	PCR multiplex	228/150	Both	171	57	122	28		8
Masood, 2013 (107)	Pakistan	Asian	LC	PB	PCR multiplex	92/150	Both	77	15	122	28		9
			PC	PB	PCR multiplex	102/150	Both	53	49	122	28		9
Matthias, 1999 (108)	Germany	Caucasian	LC	HB	PCR-RFLP	263/203	None	212	51	158	45		5
			PC	HB	PCR-RFLP	119/203	None	86	33	158	45		5
McWilliams, 2000 (109)	USA	Mixed	HNC	PB	PCR	142/109	None	118	24	89	20		6
Mondal, 2013 (110)	India	Asian	OC	HB	PCR	124/140	Both	73	51	108	32		8
Olshan, 2000 (111)	USA	Mixed	HNC	HB	PCR	172/193	Both	140	32	167	26		7
Oude Ophuis, 1998 (112)	Netherlands	Caucasian	HNC	HB	PCR	185/207	None	149	36	165	42		6
Patel, 2012 (113)	India	Asian	OC	HB	PCR	104/104	Both	48	56	55	49		8
Peters, 2006 (114)	USA	Mixed	HNC	PB	PCR	690/750	Both	568	122	588	162		8
Rao, 2017 (115)	India	Asian	OC	HB	PCR	15/15	Both	14	1	13	2		8
Reszka, 2008 (116)	Poland	Caucasian	HNC	HB	PCR-RFLP	127/145	None	120	7	141	4		5
Risch, 2003 (117)	Germany	Caucasian	LC	PB	PCR-RFLP	245/251	Both	207	38	216	35		9
Russo, 2013 (118)	Brazil	Mixed	HNC	HB	PCR-RFLP	261/514	None	142	119	275	239		6
Ruwali, 2011 (119)	India	Asian	HNC	PB	PCR multiplex	500/500	Both	365	135	397	103		9
Rydzanicz, 2005 (72)	Poland	Caucasian	HNC	PB	PCR-RFLP	182/143	None	147	35	123	20		7
Sam, 2010 (120)	India	Asian	HNC	PB	PCR	408/220	Both	331	77	204	16		9
Sánchez-Siles, 2020 (121)	Spain	Caucasian	LHC	PB	PCR-RFLP	80/23	Both	71	9	14	9		9
Saravani, 2019 (122)	Iran	Asian	OC	PB	PCR multiplex	50/63	Sex	38	12	53	10		9
Senthilkumar, 2014 (123)	India	Asian	HNC	HB	PCR	252/504	None	221	31	325	179		6
Sharma, 2006 (124)	India	Asian	OC	PB	PCR	40/87	None	23	17	74	13		7
Sikdar, 2004 (125)	India	Asian	OC	HB	PCR-RFLP	256/259	None	214	42	227	32		6
Silva, 2014 (126)	Brazil	Mixed	HNC	PB	PCR-RFLP	116/224	Both	100	16	170	54		9
Singh, 2008 (127)	India	Asian	HNC	PB	PCR-RFLP	175/198	Both	126	49	162	36		8
Singh, 2014 (128)	India	Asian	OC	PB	PCR-RFLP	122/127	None	94	28	110	17		7
Singh, 2019 (129)	India	Asian	NPC	PB	PCR-RFLP	123/189	Both	67	56	120	69		9
Soucek, 2010 (130)	Czech Republic and Poland	Caucasian	HNC	HB	PCR-RFLP	116/109	Both	92	24	93	16		7
Soya, 2007 (131)	India	Asian	HNC	HB	PCR	408/220	Both	331	77	204	16		8
Sreelekha, 2001 (132)	India	Asian	OC	PB	PCR	98/60	Both	80	18	55	5		9
Sugimura, 2006 (133)	Japan	Asian	OC	PB	PCR	122/141	None	76	46	136	105		7
Surit, 2022 (134)	India	Asian	HNC	HB	PCR	160/238	Both	131	29	217	21		8
Tata, 2022 (135)	India	Asian	OC	PB	PCR-RFLP	75/75	Both	57	18	65	10		9
To-Figueras, 2002 (136)	Spain	Caucasian	LC	PB	PCR-RFLP	204/203	None	169	35	155	48		7
Trizna, 1995 (137)	USA	Mixed	HNC	PB	PCR	127/42	Both	70	57	27	15		9
Unal, 2004 (138)	Turkey	Caucasian	LC	HB	Real-time PCR	42/89	None	25	17	64	25		6
Wei, 2012 (139)	China	Asian	NPC	HB	PCR multiplex	126/641	Both	47	79	365	276		8
Xie, 2004 (20)	USA	Mixed	OC	PB	PCR	132/143	None	93	39	101	42		7
Yadav, 2010 (21)	India	Asian	OC	PB	PCR multiplex	136/270	Both	94	42	185	85		9
Yaghmaei, 2015 (140)	Iran	Mixed	OC	PB	PCR-RFLP	35/60	Both	8	27	47	13		9
Zakiullah, 2015 (141)	Pakistan	Asian	OC	PB	Real time-PCR	200/151	Age	105	95	116	35		8
Zakiullah, 2019 (142)	Pakistan	Asian	NPC	PB	Real time-PCR and conventional PCR	130/151	Age	81	49	116	35		8

*Abbreviations* OC: oral cancer; HNC: Head and neck cancer; NPC: Nasopharyngeal cancer; LC: Laryngeal cancer; PC: Pharyngeal cancer; LHC: Laryngeal and hypopharyngeal cancers; NR: Not reported; PB: Population-based; HB: Hospital-based; PCR: Polymerase chain reaction; CTPP: Confronting two-pair primer; RFLP: Restriction fragment length polymorphism

### Meta-analysis

**Fig. 2:**
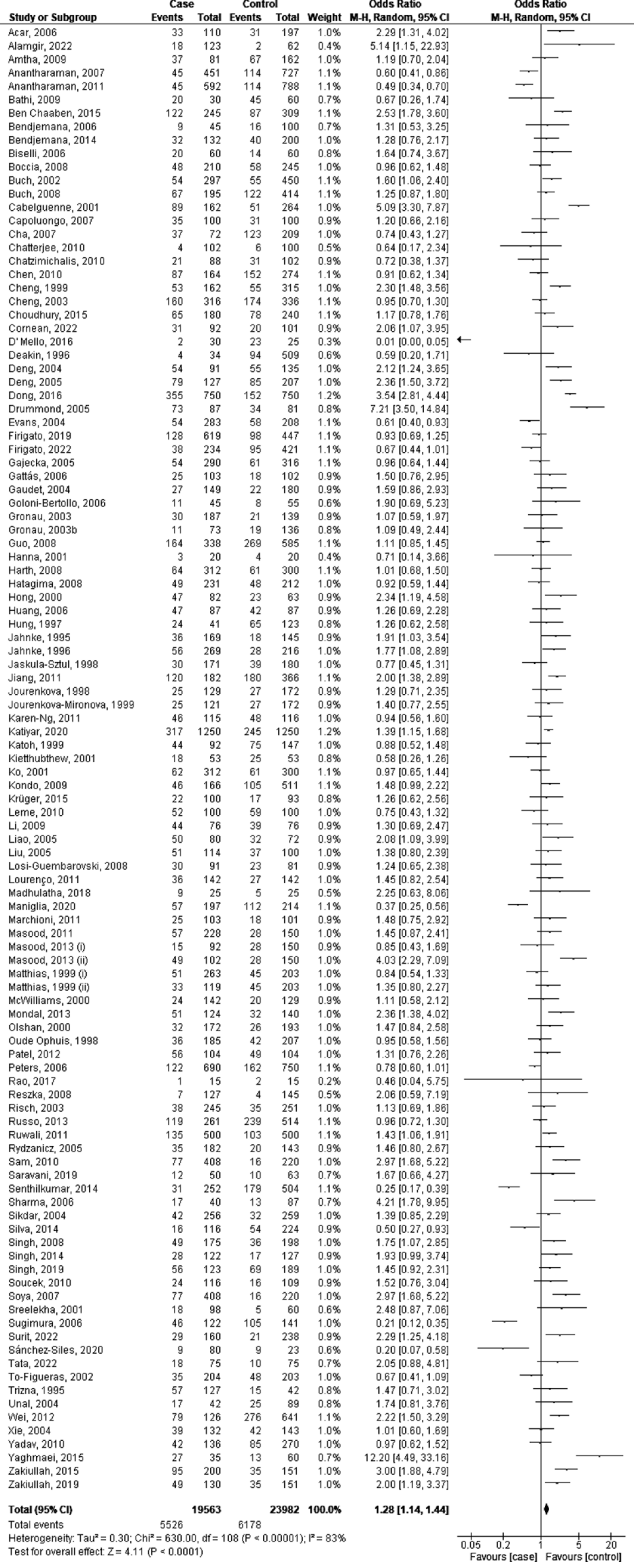
Forest plot analysis of the association between *GSTT1* polymorphism and the risk of head and neck cancer

A forest plot analysis using a random-effects model was conducted to examine the association between *GSTT1* polymorphism and the risk of HNC, as depicted in Fig. 2. The combined analysis revealed that the pooled OR was 1.28, with a 95% CI ranging from 1.14 to 1.44. This result was statistically significant with a *p*-value less than 0.0001. However, there was substantial heterogeneity among the studies, as indicated by an I^2^ value of 82%. The result suggests that there is a significant association between *GSTT1* polymorphism and the risk of HNC, with the null genotype of *GSTT1* associated with a 28% increased risk of HNC. However, due to the high heterogeneity (I^2^ = 83%), the results should be interpreted with caution as the studies included in the analysis may have varied in aspects such as study and population characteristics.

### Subgroup analysis

Table 2 presents a subgroup analysis of the association between *GSTT1* polymorphism and the risk of HNC. The subgroups are divided based on ethnicity, cancer type, sample size, control source, and quality score. In terms of ethnicity, the pooled OR was highest in Asian ethnicities (OR = 1.31), followed by Mixed (OR = 1.28), and Caucasians (OR = 1.17). For cancer type, nasopharyngeal cancer had the highest OR (1.84), followed by oral cancer with an OR of 1.20, and laryngeal cancer with an OR of 1.17. When considering sample size, studies with less than 200 samples had a higher OR (1.59) compared to those with 200 or more samples (OR = 1.23). The control source did not significantly affect the OR, with both population-based and hospital-based controls showing similar ORs of 1.29 and 1.24 respectively. The studies with a quality score of 7 or more had a higher OR (1.37) compared to those with a score of less than 7 (OR = 1.05). When considering age, the OR of 1.41 suggests a higher risk, but the high heterogeneity and *p*-value of 0.31 indicate that this result is not statistically significant. The analysis based on sex shows a significant association, with an OR of 1.36 and a *p*-value of 0.006. However, when both age and sex are considered, while the OR of 1.42 is significant, the high heterogeneity suggests caution in interpreting these results. Finally, in the group where neither age nor sex was considered, no significant association was found. These findings highlight the complexity of the relationship between the *GSTT1* null genotype and HNC risk, and how it can be influenced by factors such as age and sex. It’s important to note that all these results should be interpreted with caution due to the high heterogeneity observed in most subgroups (I^2^ > 50%).

**Table 2:** Subgroup analysis

Variable	Subgroup (*N*)	OR	95%CI		Z	*p*-value	I^2^	*P*_heterogeneity_
			Min.	Max.				
All		1.28	1.14	1.44	4.16	**< 0.0001**	82%	< 0.00001
Ethnicity								
	Asian (53)	1.31	1.09	1.58	2.83	**0.005**	87%	< 0.00001
	Caucasian (28)	1.17	0.96	1.43	1.60	0.11	71%	< 0.00001
	Mixed (28)	1.28	1.03	1.60	2.20	**0.03**	81%	< 0.00001
Cancer type								
	OC (50)	1.20	0.98	1.47	1.75	0.07	87%	< 0.00001
	NPC (12)	1.84	1.52	2.23	6.22	**< 0.00001**	59%	0.006
	LC (24)	1.17	0.90	1.52	1.16	0.25	84%	< 0.00001
Sample size								
	≥ 200 (81)	1.23	1.08	1.39	3.17	**0.002**	84%	< 0.00001
	< 200 (28)	1.59	1.09	2.32	2.40	**0.02**	76%	< 0.00001
Control source								
	PB (55)	1.29	1.08	1.53	2.87	**0.004**	86%	< 0.00001
	HB (54)	1.24	1.06	1.45	2.72	**0.007**	76%	< 0.00001
Quality score								
	≥ 7 (83)	1.37	1.20	1.56	4.61	**< 0.00001**	82%	< 0.00001
	< 7 (26)	1.05	0.82	1.35	0.40	0.69	84%	< 0.00001
Control matching								
	Age (5)	1.41	0.73	2.72	1.01	0.31	88%	< 0.00001
	Sex (8)	1.36	1.09	1.68	2.78	**0.006**	40%	0.11
	Both (54)	1.42	1.20	1.69	4.04	**< 0.0001**	84%	< 0.00001
	None (42)	1.08	0.89	1.31	0.79	0.43	80%	< 0.00001

Bolded data donate statistically significant (*p* < 0.05). *Abbreviations* OC: Oral cancer; NPC: Nasopharyngeal cancer; LC: Laryngeal cancer; PB: Population-based; HB: Hospital-based; N: Number of studies

### Meta-regression

Table 3 presents a meta-regression analysis of the variables: publication year, sample size, and quality score. For the publication year, the coefficient is ˗ 0.0003 with a *p*-value of 0.1213. For the sample size, the coefficient is -0.0002 with a *p*-value of 0.1965. For the quality score, the coefficient is 0.1283 with a *p*-value of 0.0147. In this case, only the quality score shows statistical significance as its *p*-value is less than 0.05. The results indicate that quality score increased, the effect size significantly increased.

**Table 3:** Meta-regression analysis

Variable	Coefficient	Standard error	95% lower	95% upper	Z-value	*p*-value
Publication year	˗ 0.0003	0.0002	˗ 0.0007	0.0001	˗ 1.55	0.1213
Sample size	˗ 0.0002	0.0002	˗ 0.0005	0.0001	˗ 1.29	0.1965
Quality score	0.1283	0.0526	0.0252	0.2313	2.44	**0.0147**

Bolded data donate statistically significant (*p* < 0.05)

### Sensitivity analysis

The sensitivity analysis, which included both one-study-removed and cumulative analyses, showed that the results were robust and reliable. In this case, the fact that the results did not change significantly in either analysis indicates that no single study unduly influenced the results and that the results were consistent across all studies. This adds to the validity and reliability of your findings.

*Publication bias*.

**Fig. 3:**
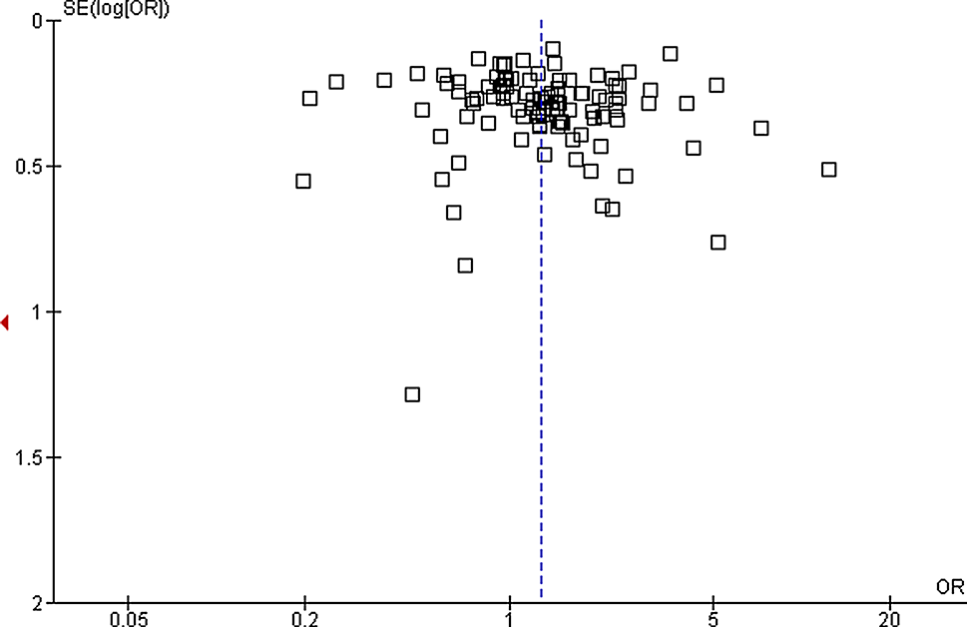
Funnel plot of the association between *GSTT1* polymorphism and the risk of head and neck cancer

Figure 3 shows the funnel plot of the association between *GSTT1* polymorphism and the risk of HNC. The *p*-values for both Egger’s test (0.895) and Begg’s test (0.108) are greater than 0.10. This suggests that there is no evidence of publication bias in the meta-analysis. Therefore, it can be interpreted that your results are likely not influenced by publication bias.

### Heterogeneity analysis

**Fig. 4:**
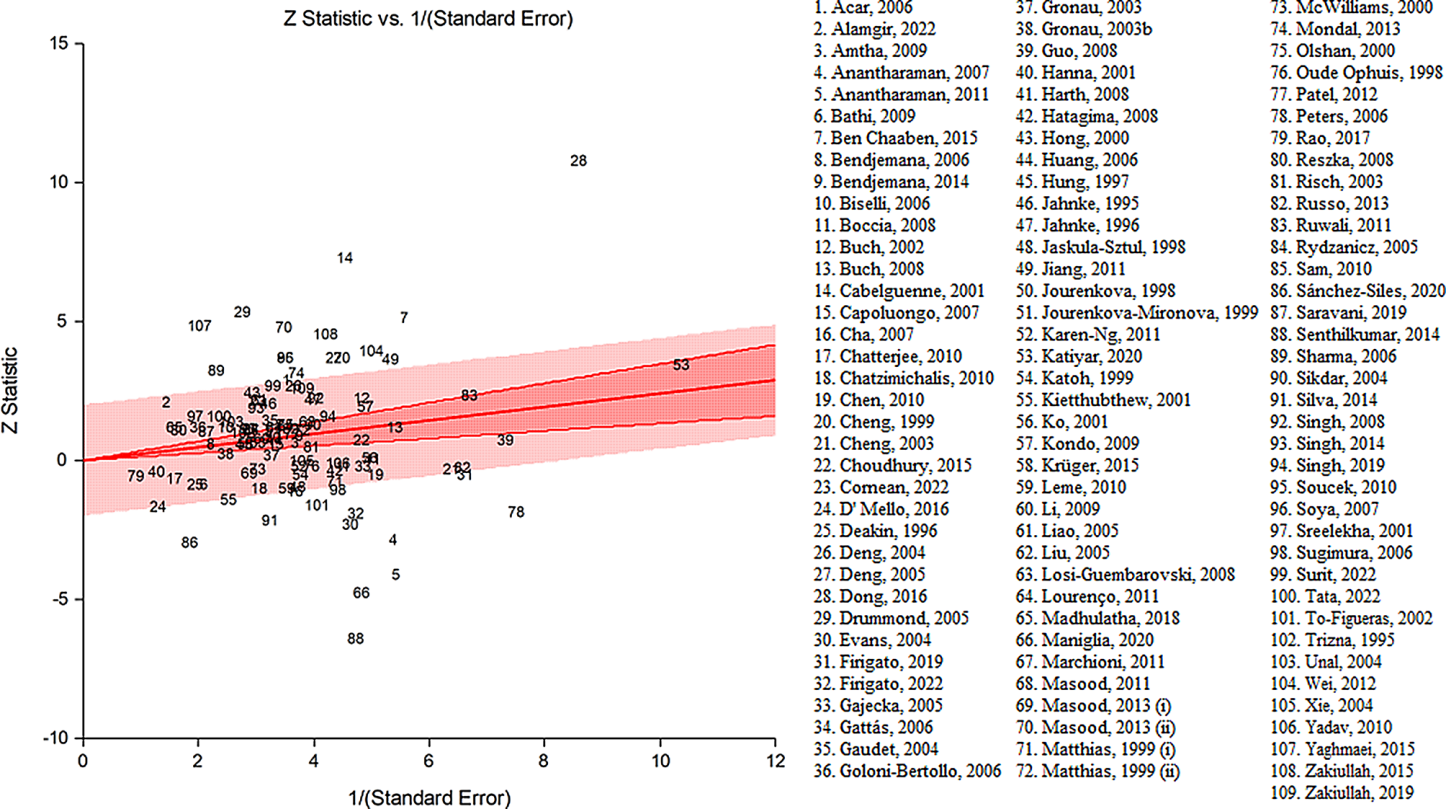
Radial plot of the association between *GSTT1* polymorphism and the risk of head and neck cancer

Figure 4 identifies the radial plot of the association between *GSTT1* polymorphism and the risk of HNC. The *p*-value of less than 0.0001 suggests that there is significant heterogeneity among the studies included in the meta-analysis. This means that there are substantial differences in the results of these studies that cannot be attributed to chance alone. The presence of outlier data in some studies could be contributing to this heterogeneity. It’s important to investigate these outliers further to understand their source and consider their impact on the overall results of the meta-analysis. Therefore, while your analysis shows a significant association between *GSTT1* polymorphism and the risk of HNC, the high heterogeneity suggests that caution should be taken when interpreting these results. Further research may be needed to explore the sources of this heterogeneity.

### TSA

**Fig. 5:**
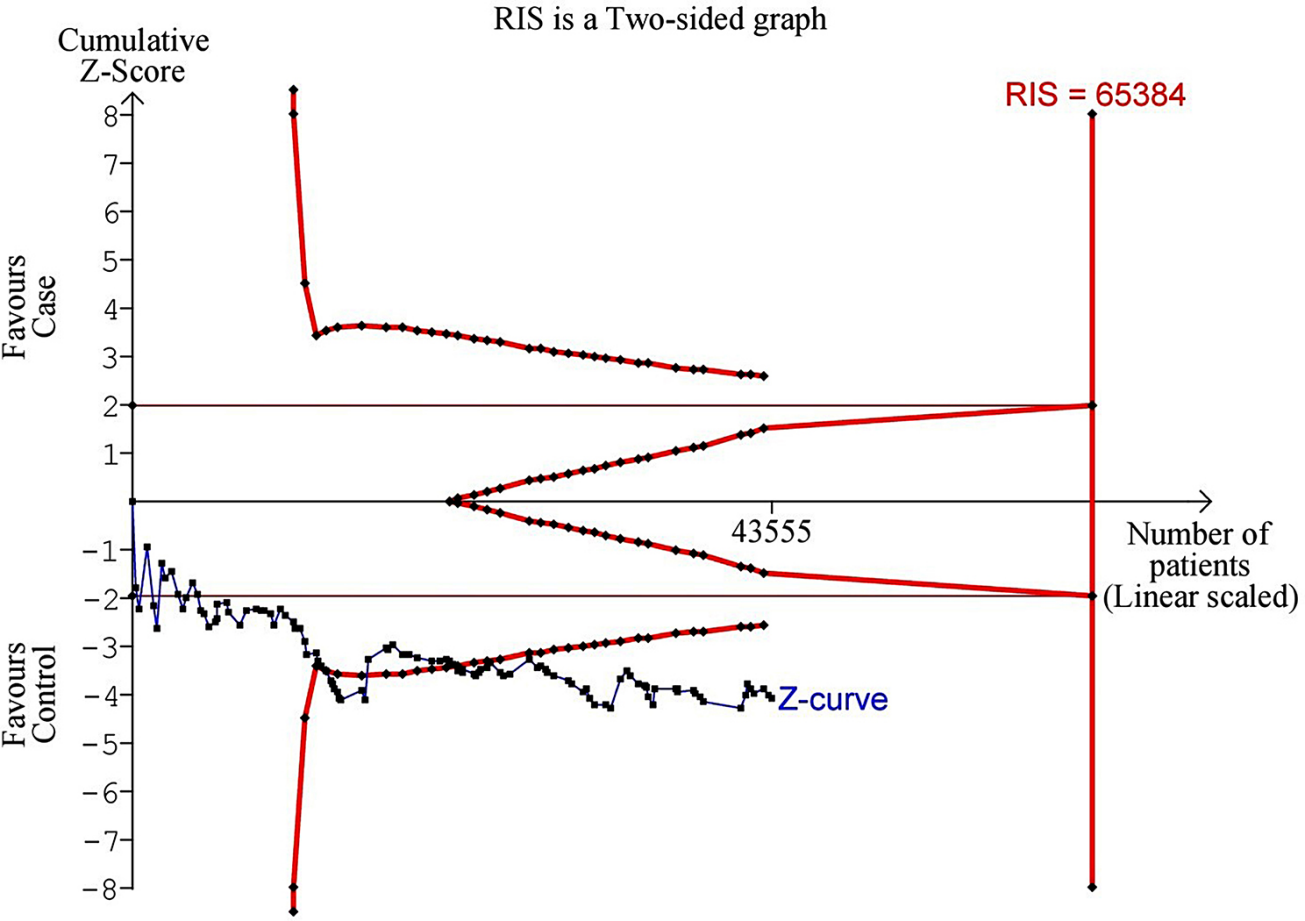
A trial sequential analysis of the association between *GSTT1* polymorphism and the risk of head and neck cancer

Figure 5 shows the TSA of the association between *GSTT1* polymorphism and the risk of HNC (D^2^ = 85%, the incidence in the intervention arm (IIA) = 28.24%; the incidence in the control arm (ICA) = 25.76%). IIA is higher than ICA. This indicates that the occurrence of the *GSTT1* null genotype under study is more frequent in the HNC group compared to the control group. The D^2^ value represents the diversity (or heterogeneity) of the study results. A high D^2^ value suggests a high degree of variability in the study results, which could be due to differences in study characteristics. The Z-curve crossing the boundary for harm suggests that the *GSTT1* polymorphism being studied may have harmful effects. However, since the number of patients in the study (43,555) is less than the RIS (65,384), the study does not have sufficient statistical power. This means that the results should be interpreted with caution as they may be prone to random errors. In other words, while the current data suggests potential harm, it does not conclusively prove it due to insufficient information size. Therefore, more research or larger studies may be needed to conclusively determine whether the *GSTT1* polymorphism is harmful.

## Discussion

A meta-analysis found that people with a certain gene variant (*GSTT1* null) had a higher risk of HNC, especially nasopharyngeal cancer. However, the studies included in the meta-analysis were very different from each other and had some limitations. Subgroup analysis revealed differences in ORs based on factors such as ethnicity, cancer type, sample size, control source, and quality score. The quality score was found to significantly impact the effect size in the meta-regression analysis. Despite these findings, the high heterogeneity and the smaller sample size compared to the RIS suggest that these results should be interpreted cautiously.

The relationship between *GSTT1* and HNC can be influenced by several factors. A study presented the results of the analysis of the joint effects or interaction between tobacco use and *GSTT1* null genotype (111). Another study (89) discovered that the *GSTT1* null genotype was linked to a higher risk among individuals who had a longer history of smoking. In addition, interaction between *GSTT1* polymorphism with other genes such as GSTs and CYPs can affect the risk of HNC (63, 129, 143, 144). The studies recommended also that occupational hazards can affect the association between *GST* polymorphisms and HNC risk (145–147). In this meta-analysis, we couldn’t check these factors due to a lack of sufficient data that future studies can check them in HNC.

GST is a family of enzymes that play an important role in detoxification by catalyzing the conjugation of many hydrophobic and electrophilic compounds with reduced glutathione (148, 149). They have been linked to the development of various cancers (150–153), but the specific role they play in HNC may require further research. The protection provided by the GSTT1 enzyme is viewed as more comprehensive, given that the gene is not only expressed in the liver but also erythrocytes. This results in a systemic action of the enzyme (154).

Research on the *GSTT1* null genotype indicates that in the United States, the absence of *GSTT1* is less prevalent compared to the *GSTM1* deletion genotype. Among individuals of European descent, it’s found that 15–31% lack a functional *GSTT1* enzyme. For African Americans, the frequencies range from 22 to 29%. Meanwhile, individuals of Hispanic origin exhibit *GSTT1* deletions at a rate of 10–12% (155–158). In terms of ethnicity, Asians are more susceptible to HNC associated with *GSTT1* null genotype, compared to their European and American counterparts (107). The present meta-analysis reported that *GSTT1* polymorphism has an association with the risk of HNC in Asians and mixed ethnicities, not Caucasians. Therefore, the prevalence of *GSTT1* null genotype may differ by geographic region (30).

In diabetic patients, the GSTM1 null genotype was found to be significantly more prevalent in the 24–36 year age group compared to other age groups (159). The present meta-analysis reported the relationship between the *GSTT1* null genotype and HNC risk, and how it can be influenced by factors such as age and sex. Therefore, outliers based on radial plot, lack of sufficient cases based on TSA, variation in age range and sex percentage can be main factors for a high heterogeneity in this meta-analysis.

The present systematic review and meta-analysis included four limitations: (1) there was substantial heterogeneity among the studies included in the meta-analysis. (2) Due to the high heterogeneity, the results should be interpreted with caution. (3) the study may not have sufficient statistical power to detect small effect sizes or rare events. (4) the study was based on published data rather than individual patient data, which may limit the ability to control for potential confounding factors. The present systematic review and meta-analysis included four strengths: (1) the study included a comprehensive analysis of 107 full-text articles, providing a broad overview of the existing literature on the association between *GSTT1* polymorphism and the risk of HNC. (2) Subgroup analysis allowed for a more nuanced understanding of how the factors might influence the association between *GSTT1* polymorphism and HNC risk. (3) Meta-regression analysis provided insights into how these variables might impact the effect size. (4) the study found no evidence of publication bias, suggesting that the results are not skewed by the selective publication of studies with positive results.

## Conclusions

This comprehensive meta-analysis revealed a significant association between the *GSTT1* null genotype and an increased risk of HNC, with variations based on factors such as ethnicity, cancer type, sample size, control source, and quality score. Despite the robustness of the results, there was high heterogeneity among studies and limited statistical power due to a smaller number of cases. From a clinical perspective, these findings underscore the potential of the *GSTT1* null genotype as a genetic marker for HNC susceptibility, which could have significant implications for early detection and prevention strategies. However, further research is needed to confirm these findings and elucidate the underlying mechanisms. This study sets the stage for future research in this area, highlighting the importance of considering factors such as ethnicity, cancer type, sample size, control source, and quality score in understanding the complex relationship between *GSTT1* null genotype and HNC risk.

### Electronic supplementary material

Below is the link to the electronic supplementary material.

Supplementary Material 1

## Data Availability

All data generated or analyzed during this study are included in this published article and its Supplementary information files.
