# Supplementary material for: Glutathione S-transferase theta 1 (GSTT1) deletion polymorphism and susceptibility to head and neck carcinoma: a systematic review with five analyses
Source: BMC Cancer. 2024 Jul 22;24:885. doi: 10.1186/s12885-024-12618-7 (PMC11264357; doi:10.1186/s12885-024-12618-7)
Supplement: Supplementary file 1 — Supplementary Material 1 [file 12885_2024_12618_MOESM1_ESM.docx]

**Search Strategy**

**PubMed**: ("GSTT1"[Title/Abstract] OR "GST"[Title/Abstract] OR "GSTP1"[Title/Abstract] OR "Glutathione S-transferase"[Title/Abstract]) AND (“mouth”[Title/Abstract] OR “OSCC”[Title/Abstract] OR “oral”[Title/Abstract] OR “tongue”[Title/Abstract] OR “head and neck”[Title/Abstract] OR “HNSCC“[Title/Abstract] OR “nasopharyngeal”[Title/Abstract] OR “nasopharynx” [Title/Abstract] OR “oropharyngeal”[Title/Abstract] OR “salivary gland”[Title/Abstract] OR “laryngeal”[Title/Abstract] OR “larynx” [Title/Abstract] OR “hypopharyngeal”[Title/Abstract] OR “pharyngeal”[Title/Abstract] OR “pharynx”[Title/Abstract] OR “oral cavity”[Title/Abstract] OR “hypopharynx” [Title/Abstract]) AND (tumor*[Title/Abstract] OR carcinoma*[Title/Abstract] OR cancer*[Title/Abstract] OR neoplasm*[Title/Abstract]) AND (“polymorphism*”[Title/Abstract] OR “variant*”[Title/Abstract] OR “gene*” [Title/Abstract] OR “genotype*” [Title/Abstract] OR “allele*” [Title/Abstract])

**Scopus**: (TITLE-ABS-KEY (“GSTT1”) OR TITLE-ABS-KEY (“GSTP1”) OR TITLE-ABS-KEY (“GST”) OR TITLE-ABS-KEY(“Glutathione S-transferase”)) AND (TITLE-ABS-KEY (“mouth”) OR TITLE-ABS-KEY (“OSCC”) OR TITLE-ABS-KEY (“oral”) OR TITLE-ABS-KEY (“tongue”) OR TITLE-ABS-KEY (“head and neck”) OR TITLE-ABS-KEY (“HNSCC“) OR TITLE-ABS-KEY (“nasopharyngeal”) OR TITLE-ABS-KEY (“nasopharynx”) OR TITLE-ABS-KEY (“oropharyngeal”) OR TITLE-ABS-KEY (“Salivary gland”) OR TITLE-ABS-KEY (“laryngeal”) OR TITLE-ABS-KEY (“larynx”) OR TITLE-ABS-KEY (“hypopharyngeal”) OR TITLE-ABS-KEY (“pharyngeal”) OR TITLE-ABS-KEY (“pharynx”) OR TITLE-ABS-KEY (“oral cavity”) OR TITLE-ABS-KEY (“hypopharynx”)) AND (TITLE-ABS-KEY (tumor*) OR TITLE-ABS-KEY (carcinoma*) OR TITLE-ABS-KEY (cancer*) OR TITLE-ABS-KEY (neoplasm*)) AND (TITLE-ABS-KEY (“polymorphism*”) OR TITLE-ABS-KEY (“variant*”) OR TITLE-ABS-KEY (“gene*”) OR TITLE-ABS-KEY (“genotype*”) OR TITLE-ABS-KEY (“allele*”))

**Web of Science**: TS=(“GSTT1” OR “GSTP1” OR “GST” OR “Glutathione S-transferase”) AND TS=(“mouth” OR “OSCC” OR “oral” OR “tongue” OR “head and neck” OR “HNSCC“ OR “nasopharyngeal” OR “nasopharynx” OR “oropharyngeal” OR “salivary gland” OR “laryngeal” OR “larynx” OR “hypopharyngeal” OR “pharyngeal” OR “pharynx” OR “oral cavity” OR “hypopharynx”) AND TS=(tumor* OR carcinoma* OR cancer* OR neoplasm*) AND TS=(“polymorphism*” OR “variant*” OR “gene*” OR “genotype*” OR “allele*”)

**Cochrane Library**: (“GSTT1”:ti,ab,kw OR “GSTP1”:ti,ab,kw OR “GST”:ti,ab,kw OR “Glutathione S-transferase”:ti,ab,kw) AND (“mouth”:ti,ab,kw OR “OSCC”:ti,ab,kw OR “oral”:ti,ab,kw OR “tongue”:ti,ab,kw OR “head and neck”:ti,ab,kw OR “HNSCC“:ti,ab,kw OR “nasopharyngeal”:ti,ab,kw OR “nasopharynx”:ti,ab,kw OR “oropharyngeal”:ti,ab,kw OR “salivary gland”:ti,ab,kw OR “laryngeal”:ti,ab,kw OR “larynx”:ti,ab,kw OR “hypopharyngeal”:ti,ab,kw OR “pharyngeal”:ti,ab,kw OR “pharynx”:ti,ab,kw OR “oral cavity”:ti,ab,kw OR “hypopharynx”:ti,ab,kw) AND (tumor*:ti,ab,kw OR carcinoma*:ti,ab,kw OR cancer*:ti,ab,kw OR neoplasm*:ti,ab,kw) AND (“polymorphism*”:ti,ab,kw OR “variant*”:ti,ab,kw OR “gene*”:ti,ab,kw OR “genotype*”:ti,ab,kw OR “allele*”:ti,ab,kw)


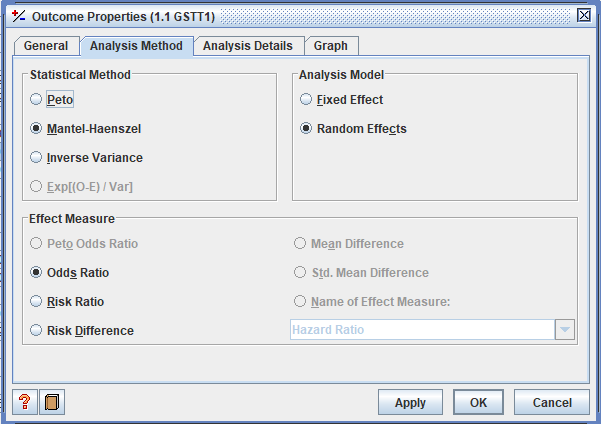


Supplement Figure 1. Options selected in Review Manager.


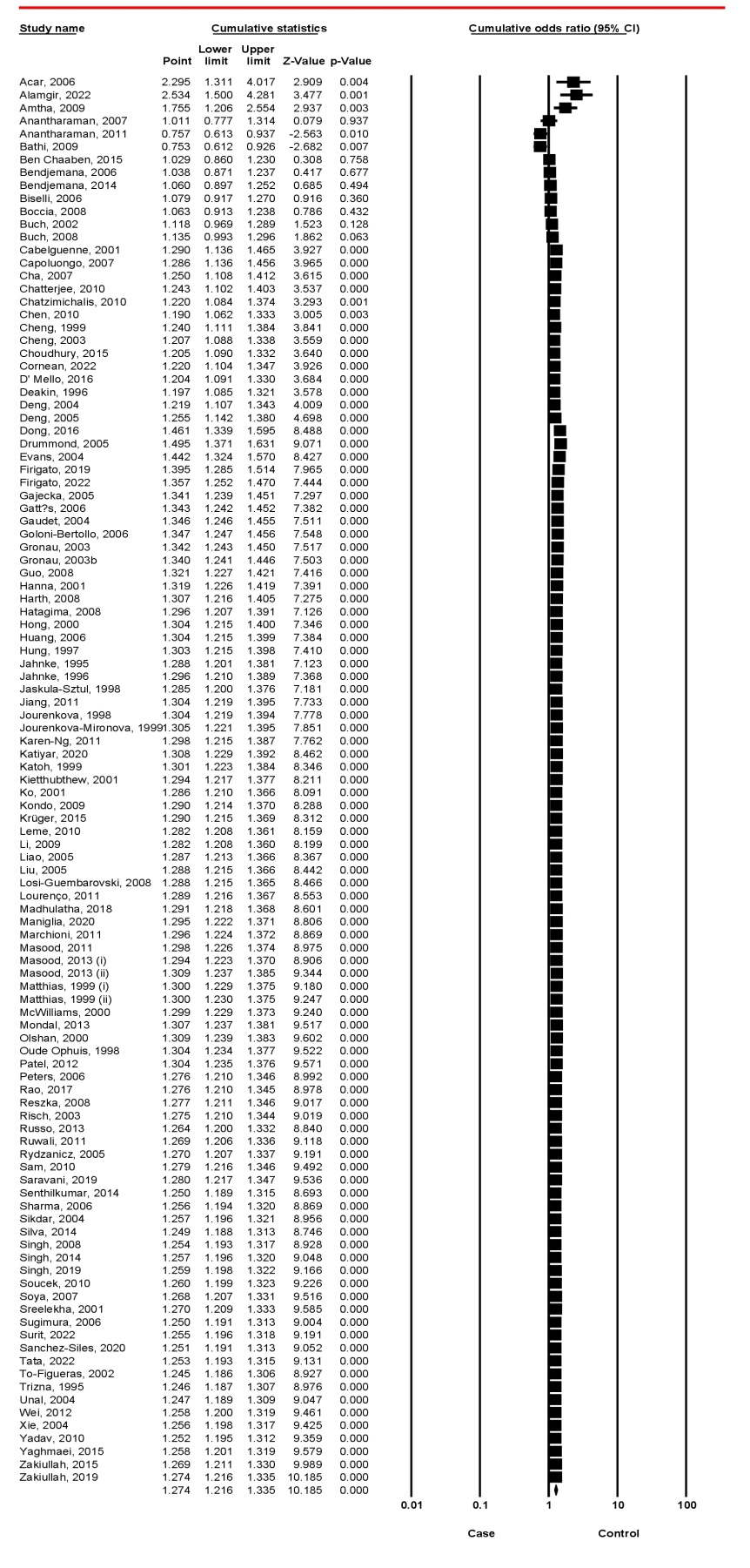


Supplement Figure 2. Cumulative analysis


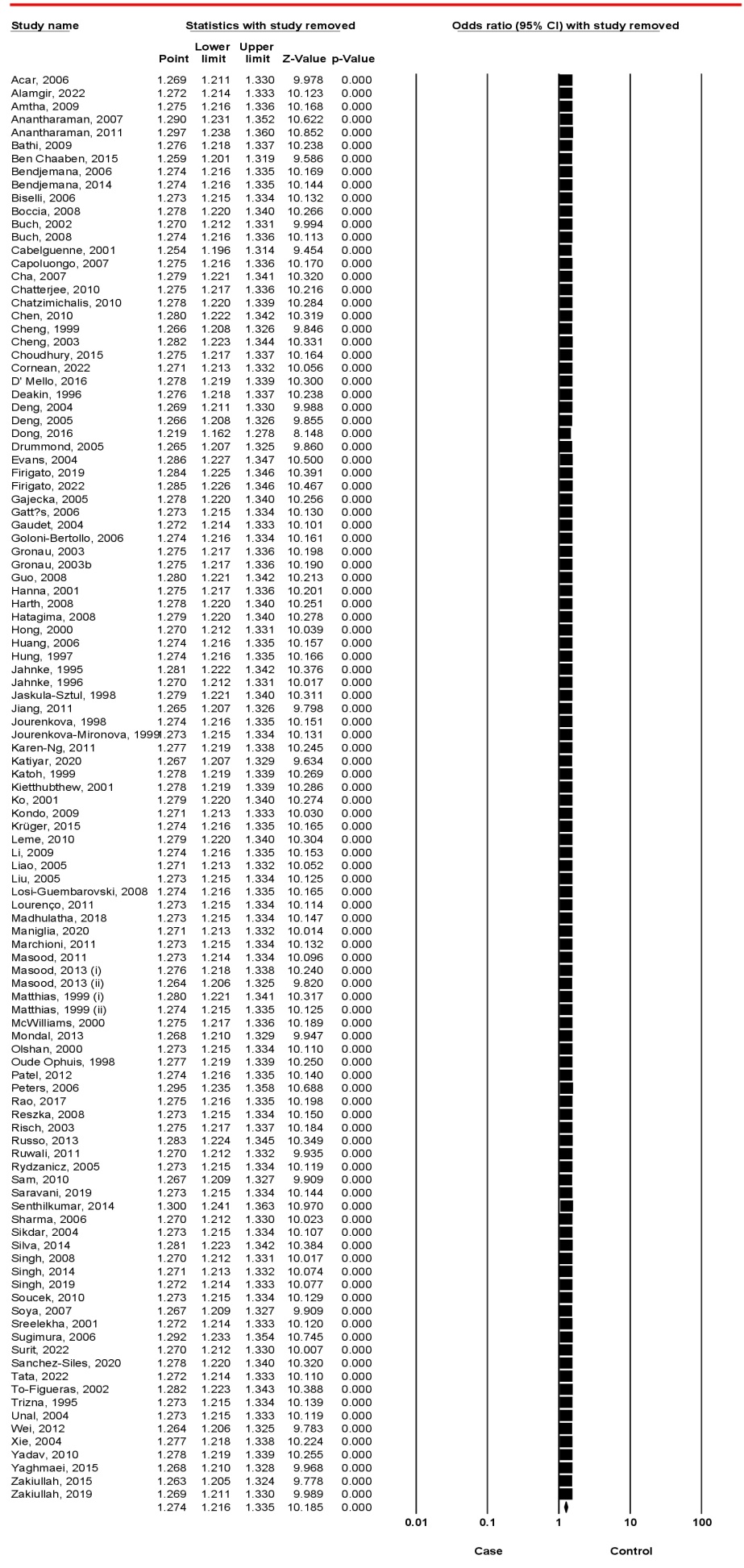


Supplement Figure 3. One-study-removed analysis


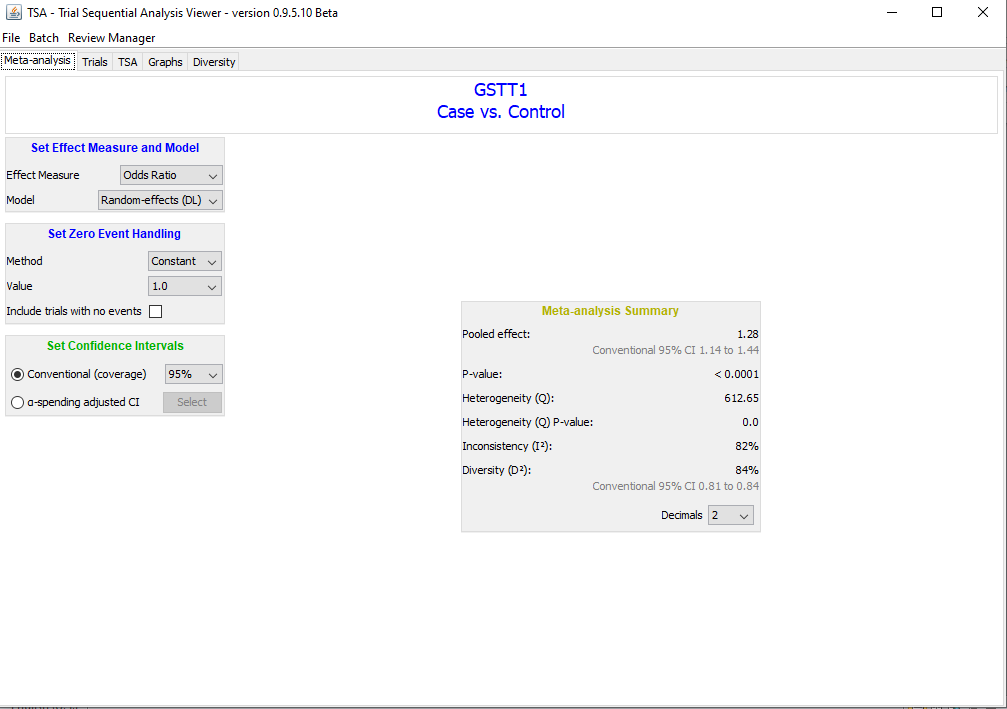


Supplement Figure 4. Selected options and summary data from trial sequential analysis


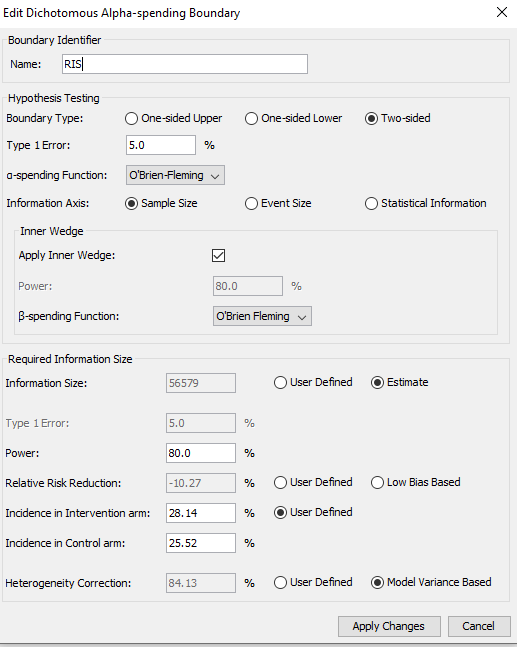


Supplement Figure 5. Alpha-spending Boundary in trial sequential analysis
